# Comparison of Patient-Controlled versus Continuous Epidural Analgesia in Adult Surgical Patients: A Systematic Review

**DOI:** 10.3390/jcm12093164

**Published:** 2023-04-27

**Authors:** Ganapathy van Samkar, Yan Ru Tan, Henning Hermanns, Benedikt Preckel, Faridi S. Jamaludin, Markus W. Hollmann, Markus F. Stevens

**Affiliations:** 1 Department of Anesthesiology, Amsterdam UMC, Location AMC, University of Amsterdam, 1105 AZ Amsterdam, The Netherlands; g.vansamkar@amsterdamumc.nl (G.v.S.);; 2 Department of Anesthesiology, Singapore General Hospital, Outram Road, Singapore 169856, Singapore; tan.yan.ru@singhealth.com.sg; 3 Medical Library AMC, Amsterdam UMC, University of Amsterdam, 1105 AZ Amsterdam, The Netherlands

**Keywords:** patient-controlled epidural analgesia, continuous epidural analgesia, surgery

## Abstract

Background: The advantages of PCEA over CEA have been demonstrated in obstetric patients. Whether a similar benefit applies to surgical patients is unclear. Methods: Embase, PubMed, and Cochrane Library were searched, enabling a systematic review of studies comparing PCEA and CEA in adult surgical patients (PROSPERO: CRD42018106644). The study quality was assessed using the Cochrane risk-of-bias tool (RoB2). The primary outcome was pain scores on postoperative day one (POD1). Secondary outcomes were 24 or 48 h epidural or intravenous total analgesic dose, systemic analgesics, manual top-ups, side effects, and patient satisfaction. Results: Six randomized controlled trials with high heterogeneity of study characteristics were identified with a moderate risk of bias. Two studies showed significantly reduced resting pain scores on POD1 in PCEA compared with CEA patients (36–44%, *p* < 0.05). Four studies found comparable pain scores between these groups. PCEA use reduced epidural medication (28% to 40% reduction, *p* < 0.01) in four studies. One study found a 23% reduction (*p* < 0.001) of top-ups in PCEA; intravenous morphine use by PCEA patients was reduced (0.16 vs. 3.45 mg per patient, *p* < 0.05) in one study. PCEA patients were more satisfied with analgesia (*p* < 0.001) in two studies. Nausea and vomiting were reduced in PCEA (*p* = 0.01). Conclusions: Regarding the reduction in pain scores, the effects of PCEA were not significant or clinically not relevant. However, regarding the amount of epidural drug use, the amount of required rescue systemic analgesics, patient satisfaction, and the number of required top-ups, PCEA had advantages over CEA in surgical patients.

## 1. Introduction

Epidural analgesia is still considered the standard of care for major upper abdominal or thoracic surgery [1,2,3,4]. However, epidural analgesia is known to have a failure rate of approximately 30% and therefore requires frequent epidural top-ups and/or systemic analgesic rescue medication [5].

During labor, the superiority of patient-controlled epidural analgesia (PCEA) compared with continuous epidural analgesia (CEA) has been proven in numerous clinical studies and has been confirmed in systematic reviews with meta-analyses. The more novel method of PCEA used programmed intermittent mandatory bolus (PIEB) [6,7,8,9]. An earlier meta-analysis of studies comparing PCEA (without background infusion but only patient boluses) with CEA also found advantages of PCEA in labor analgesia [10]. The implementation of PCEA during labor resulted in reduced drug requirements, reduced frequency of motor weakness, and reduced top-ups when compared to CEA. PCEA is most commonly implemented by manual patient-initiated boluses of local anesthetic on top of a baseline infusion. One of the factors involved in the inferiority of constant epidural infusion may be a progressive regression of the block. A higher infusion rate is associated with more usage of local anesthetic and more maternal block in obstetric analgesia, possibly contributing to a higher rate of instrumental deliveries [8,10]. The additional cost of purchasing special pumps and training personnel is justified if PCEA offers advantages to the patient. However, the efficacy of PCEA in a surgical (non-obstetric) population has been investigated less frequently, without published systematic reviews or meta-analyses. The results from a relatively homogenous population of young and healthy females with a uniform indication for short-term peripartum analgesia may not be applicable to a totally different population including males or the elderly, or in cases including higher ASA categories and concomitant medication for a variety of operations requiring a longer stay in hospital. We performed a systematic review of studies comparing PCEA and CEA in the adult population undergoing non-cardiac and non-obstetric surgery to examine evidence pertaining to (1) pain scores; (2) the total amount of epidural and intravenous medication used over 24 or 48 h; (3) the number of manual top-ups required; (4) the use of additional systemic analgesics; (5) side effects; and (6) patient satisfaction.

Our hypothesis, based on evidence in the obstetric population, was that the use of PCEA leads to reduced pain scores in rest and movement compared with CEA. Furthermore, we expected a reduction in the use of epidural medication, a reduction in top-ups, and a reduction in the use of systemic analgesics, with improved patient satisfaction in the PCEA group. Our aim was to investigate and compare the existing methods of epidural analgesia in non-obstetric patients undergoing surgery. Our secondary aim was to ascertain which method offers the best analgesic benefit and fewest side effects.

## 2. Methods

The review protocol was registered in the International Prospective Register of Systematic Reviews (PROSPERO) with registration number CRD42018106644. The guidelines of the Preferred Reporting Items for Systematic Reviews and Meta-Analyses (PRISMA) were followed [11]. Embase (Ovid), PubMed, and the Cochrane Library were searched for studies performed before 18 May 2022, to identify relevant trials. The search terms and search strategy are described in Appendix A.

### 2.1. Inclusion Criteria

Adult surgical patients receiving perioperative epidural analgesia were included. The intervention involved patient-controlled epidural analgesia (PCEA, with or without continuous background infusion), and for comparison, conventional CEA was considered. Our primary outcome was postoperative pain during rest on day 1 (visual analog scores or comparable scores and/or numeric rating scale). The secondary outcomes were (1) postoperative pain during movement on day 1; (2) the amount of epidural medication used; (3) the number of top-ups required; (4) the use of systemic analgesics; and (5) patient satisfaction. To enable a better comparison with obstetric studies that have a relatively short duration of epidural treatment, we chose day 1 pain scores as the primary endpoint. Furthermore, the first postoperative day is often the day with the highest pain score, and most studies include the pain score on day 1 in their analysis.

### 2.2. Exclusion Criteria

Studies involving patients aged <18 years, obstetric patients during labor, language not native to at least two team members (Chinese, Spanish, French, Russian, Korean, and Japanese), conference abstracts or communications, comparisons using PCEA followed by CEA or vice versa in the same patient, the use of programmed intermittent epidural bolus instead of PCEA, and publications other than a randomized controlled trial (RCT) or cohort analysis were excluded from this study. We included cohort analyses to enable a more complete comparison in case there was an insufficient number of RCTs found in the search.

Titles and/or abstracts of studies retrieved using the search strategy were independently screened on Rayyan.qcri.org by two review authors (G.v.S. and Y.R.T.) to identify studies that met the inclusion criteria. Conflicts in this stage were resolved by a third reviewer (M.F.S.). The full text of potentially eligible studies was retrieved and independently assessed for eligibility by two review authors (G.v.S. and Y.R.T.). Any disagreement between them over the eligibility of particular studies was resolved through discussion with a third reviewer (M.F.S.). After the final selection of studies to be included, a predefined data extraction form was used (by G.v.S. and Y.R.T.) to extract data from the respective studies for assessment of study quality and evidence synthesis (Appendix B). The risk of bias in the included studies was assessed according to the guidelines of the Cochrane Collaboration, using the Cochrane risk-of-bias tool (RoB2) and Cochrane’s Review Manager version 5.4 [12,13].

### 2.3. Meta-Analysis

Meta-analysis of data was performed using Cochrane’s Review Manager 5.4 software when four or more RCTs were available for a specific outcome. Differences in primary and secondary outcome parameters were expressed as mean differences and standard deviations. We assessed statistical heterogeneity using Cochrane’s Q statistic (with *p* < 0.05 considered significant) and expressed the quantity using the I2 statistic and 95% confidence interval (CI). We followed the Cochrane Handbook’s classification for the importance of I2. Between-study variance (Tau^2^) and the I^2^ statistic were computed to estimate the percentage of variability in effect sizes that cannot be explained by sampling errors. We defined statistical heterogeneity as high when I^2^ > 50. Forest plots were used to present the data when appropriate for specific outcomes.

We performed a subanalysis specifically related to the type of surgery if two or more studies were retrieved in patients undergoing the same type of surgery.

## 3. Results

**Figure 1:**
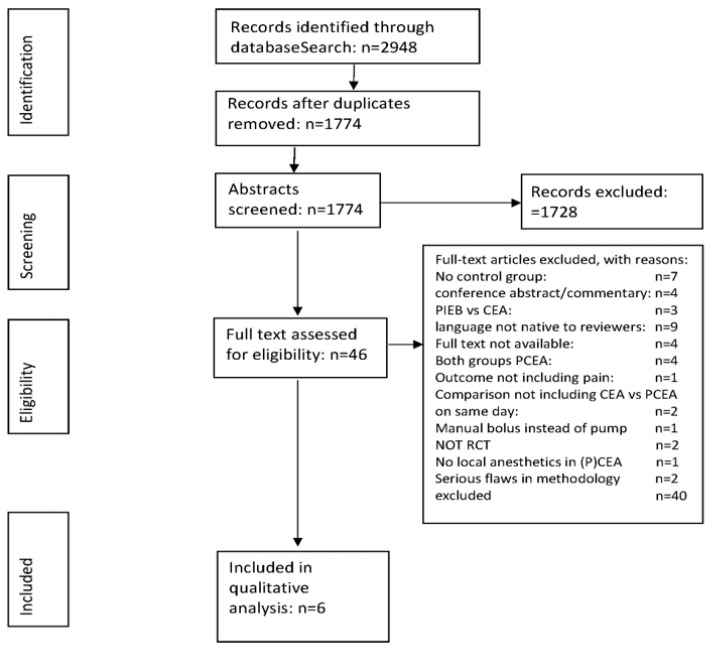
Study selection flow diagram.

The systematic search of the literature before 18 May 2022 yielded a total of 2948 studies. The PRISMA study selection flow diagram is depicted in Figure 1. After the removal of duplicates, 1774 studies were screened for titles and abstracts. Initially, we selected 11 studies, including 1687 patients [14,15,16,17,18,19,20,21,22,23,24]. To improve homogeneity for the final analysis, we decided to include only randomized controlled studies without serious flaws in methodology and studies using epidural analgesia containing local anesthetic in protocols of PCEA (including the boluses) and CEA. The final selection comprised 6 studies, including 480 patients. One study had a low risk of bias, and five studies had an unclear risk of bias.

**Figure 2:**
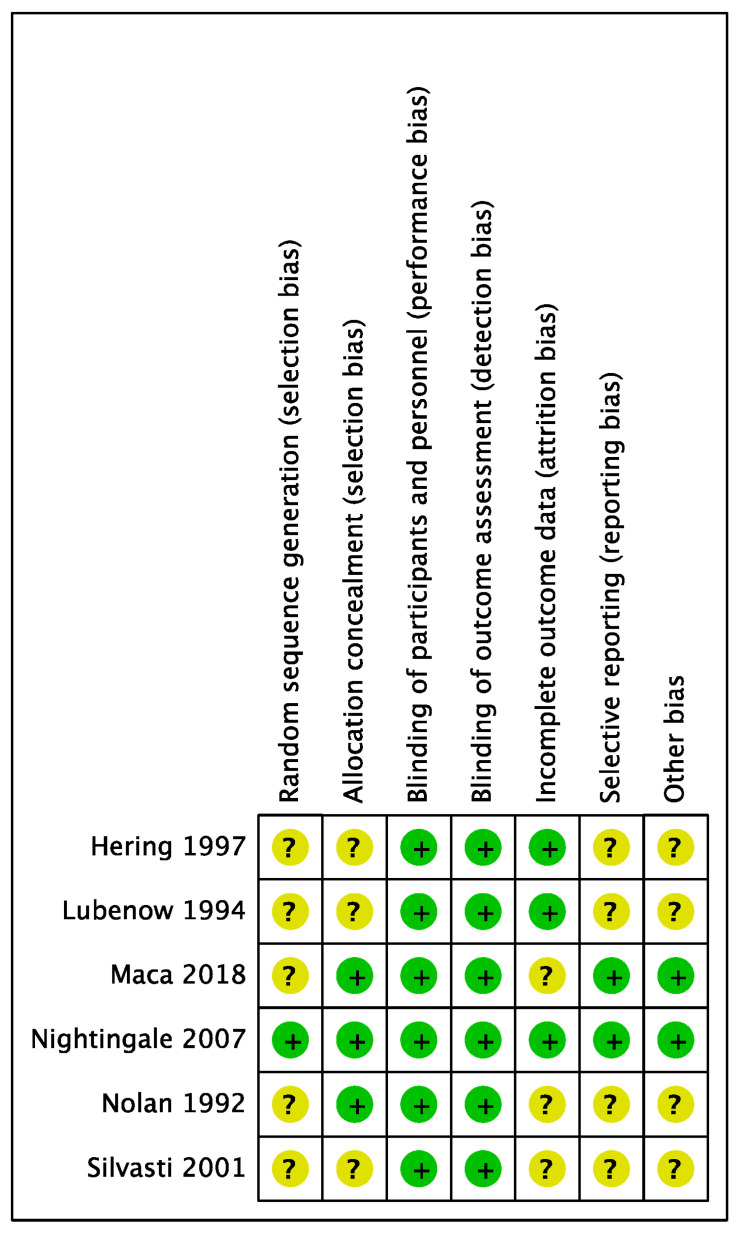
Risk-of-bias assessment. Green signifies a low risk of bias, and yellow is an unclear risk of bias. Hering [21], Lubenow [17], Maca [20], Nightingale [24], Nolan [16], Silvasti [22].

The reasons for exclusion are specified in Figure 1. The risk-of-bias assessment is specified in Figure 2.

Baseline characteristics (type of surgery, patient population, location of epidural, and PCEA/CEA regimen) are described in Table 1. The studies were heterogeneous in nature, including colonic surgery, total knee replacement, total hip replacement, pelvic surgery, major abdominal surgery, and urological, orthopedic, or thoracic surgery. ASA categories of the included patients ranged from I to III. The mean age of the included patients ranged from 33 to 74 years. The levels of insertion of the epidural catheter varied from T10 to L4. The epidural solutions used were bupivacaine (0.1–0.125%) or levobupivacaine (0.1%), with or without the addition of opiates (fentanyl 1–10 micrograms/mL or sufentanil 1 microgram/mL). The continuous infusion rate of PCEA regimens varied from 3 to 8 mL/h, and the bolus rates varied from 1 to 5 mL/bolus.

### 3.1. Primary Endpoint

Pain scores: All studies reported resting pain scores. Two of the six studies we included in the analysis found significantly reduced pain scores on the first postoperative day (POD1) in patients treated with PCEA (Table 2) [17,24]: Pain scores were reduced by 36–44% in PCEA patients, compared with CEA-treated patients. Three studies provided information about pain scores during motion [21,22,24]. One study revealed that PCEA-treated patients had a significant reduction (*p* < 0.001) in pain scores during motion, compared with CEA-treated patients [21]. However, a different study found the exact opposite: reduced VAS pain scores in CEA-treated patients during motion (1.9 vs. 3.4, *p* < 0.01) [22]. Nightingale et al. did not use the visual analog scale (1–10) but used the four-point verbal rating scale (VRS) by Wessex (0 = no pain, 1 = mild, 2 = moderate, and 3 = severe) instead. They then presented the area under the curve (AUC) for pain (PCEA 15.6 vs. CEA 32, *p* < 0.001). We calculated the means and standard deviations of the Wessex VRS score from the original diagram with confidence intervals by Nightingale et al. to enable a better comparison, with a 1–10 score as used by other authors [24] (sqrt of the number of patients x upper-lower boundary of 95% confidence interval/3.92) [24].

We found an insufficient number of studies investigating comparable types of surgery to perform a proper meta-analysis. We present the results in forest plots as Appendix A, Appendix B, Appendix C, Appendix D, Appendix E, Appendix F, Appendix G.

**Figure A1:**
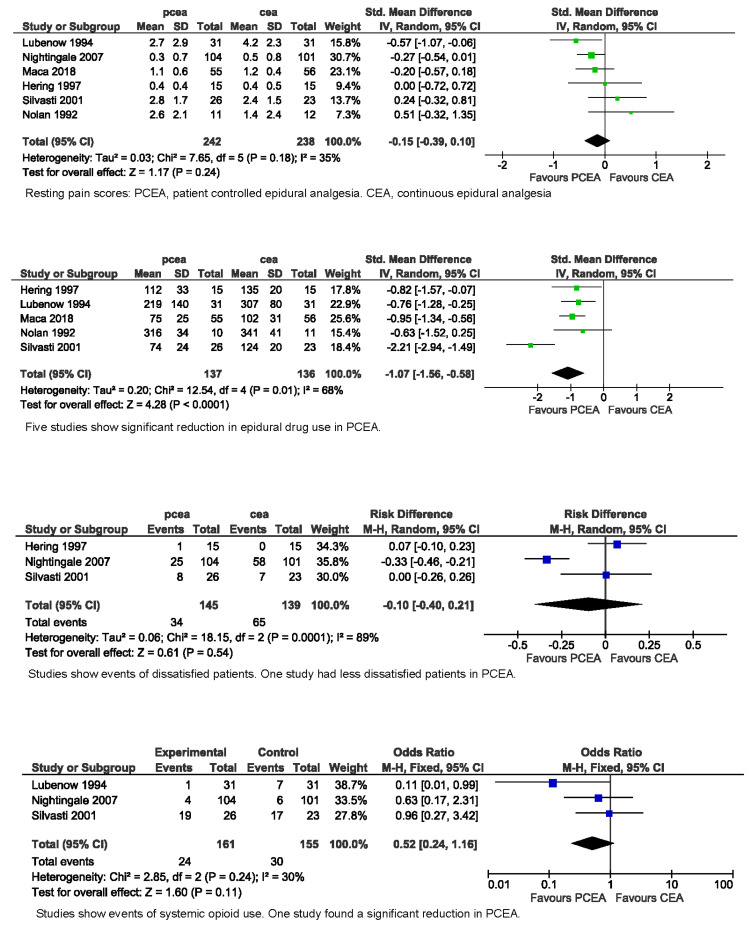
Forest plots of studies comparing PCEA vs. CEA, primary and secondary endpoints. Hering [21], Lubenow [17], Maca [20], Nightingale [24], Nolan [16], Silvasti [22].

The forest plot of resting pain scores (Figure A1 in Appendix C) is arranged in order of studies favoring PCEA followed by studies favoring CEA and suggests different effect sizes in the different types of populations. There is some degree of heterogeneity, signified by Tau^2^ (0.03) and I^2^ (35%). 

### 3.2. Secondary Endpoints

Epidural medication: Four out of six studies found a significant reduction in epidural drug use by 28–40% in PCEA patients (*p* < 0.01) [17,20,21,22].

Top-ups: One study found a 23% reduction (*p* < 0.001) in epidural top-ups among PCEA patients [24].

Patient satisfaction: In two studies, the percentage of good patient satisfaction was higher in PCEA patients (76% PCEA vs. 43% CEA, and a Likert score of 4.3 PCEA vs. 2.8 CEA, *p* < 0.001) [20,24]. One other study did not find a significant difference in patient satisfaction between the two groups; however, both groups in this study showed a satisfaction percentage of 90% or higher [21].

Use of intravenous rescue opioid medication: One study found a reduced requirement of intravenous morphine (mean dose per patient: 0.16 vs. 3.45 mg, *p* < 0.05) in PCEA patients [17]. Two other studies also found comparable but less pronounced differences between the two groups (NS) [22,24].

We examined the differences between PCEA and CEA regarding the outcome parameters concerning safety, namely hypotension, respiratory depression, and systemic opioid use, during a 24 h follow-up period.

Hypotension was described by two authors, but no significant differences between the groups were found (*p* = 0.78) [20,22].

Respiratory depression was described in one study, in 8/55 patients in the PCEA group versus 15/56 patients in the CEA group, but no significant differences between these groups were found (*p* = 0.12) [20].

Systemic opioid use was described in three studies, but no significant differences between the groups were found in the pooled analysis (*p* = 0.11) [17,22,24].

Other side effects:

Nausea and vomiting (PONV) were described in four studies. In PCEA, this was less frequent, with an odds ratio of 0.31 (95% CI 0.12–0.76), *p* = 0.01 [16,17,21,22].

Itching was described in three studies, but no significant differences between the groups were found (*p* = 0.70) [16,17,21].

Urine retention was described in two studies without significant differences between the groups (*p* = 0.10) [21,22].

**Figure A2:**
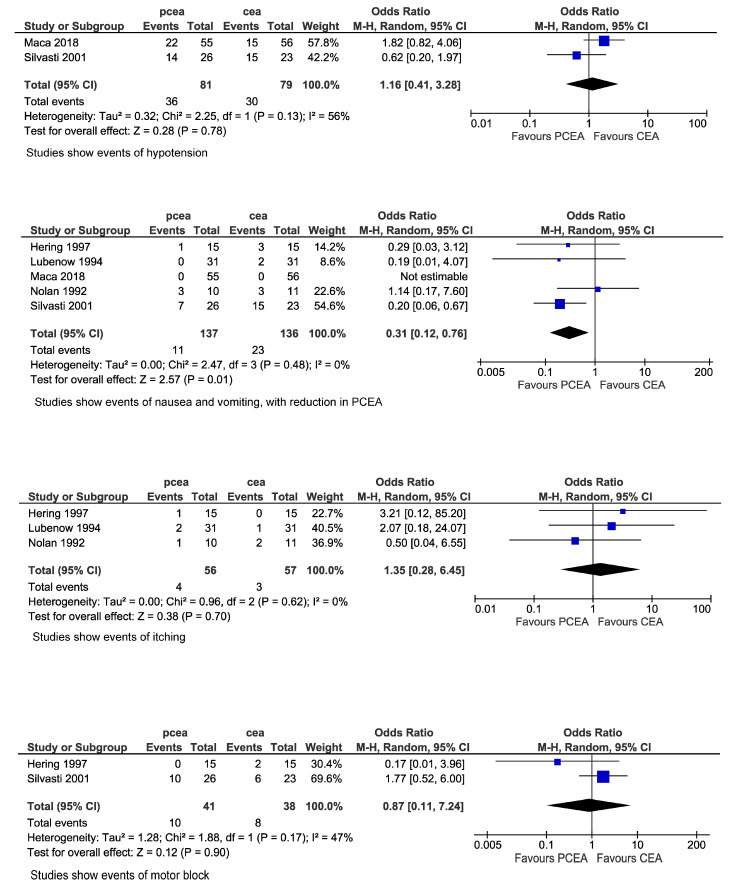
Safety profiles and side-effects. Hering [21], Lubenow [17], Maca [20], Nightingale [24], Nolan [16], Silvasti [22].

Motor block was described by Hering and Silvasti, without significant differences between the groups (*p* = 0.90) [21,22]. Forest plots of the outcomes are presented in Figure A2 in Appendix D. 

**Figure A3:**
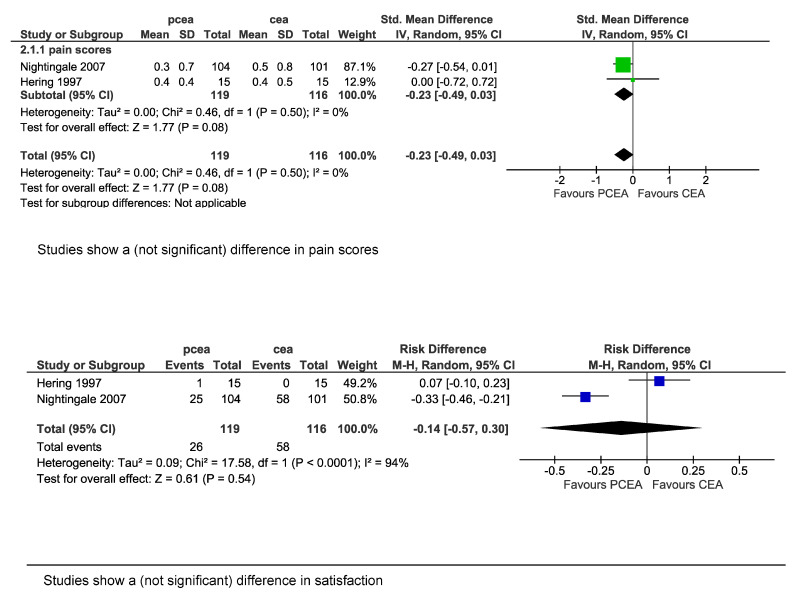
Subanalysis abdominal surgery. Hering [21], Nightingale [24].

Subanalysis of abdominal operations: Hering et al. and Nightingale et al. performed studies involving patients undergoing abdominal surgery [21,24]. We compared the two studies to be able to draw possible conclusions in comparable operations (forest plot of outcomes in Figure A3 in Appendix E). The pooled studies revealed a (non-significant) difference in pain scores (*p* = 0.08). Top-ups, described by Nightingale et al., were reduced in PCEA (*p* < 0.005). Epidural medication needs, described by Hering et al., were reduced in PCEA (*p* = 0.03). The pooled comparisons of patient satisfaction, systemic opioid use, itching, motor block, and nausea and vomiting did not reveal any significant differences between PCEA and CEA.

The funnel plot illustrates the extent of publication bias: It is not funnel-shaped but reasonably symmetrical. Thus, the low number of studies does not show detectable bias (Appendix F).

A summary of findings is found in Appendix G.

## 4. Discussion

We set out to find evidence of the potential advantages of PCEA in surgical patients. Our findings were as follows:

(1) PCEA significantly reduced resting pain scores in some studies, while other studies found no difference; (2) PCEA reduced the dose of epidural medication; (3) PCEA reduced the need for systemic medication; (4) PCEA reduced the requirement for additional top-ups; (5) PCEA increased patient satisfaction; (6) PCEA reduced the frequency of nausea and vomiting.

The quality of recovery is measured using a multidimensional assessment, which includes pain, nausea and vomiting, sleep quality, and satisfaction [25,26]. We did not perform a multidimensional measurement, but we assessed several independent factors affecting the quality of recovery.

Regarding the pain scores, it is noteworthy that Hering et al. found that patients receiving PCEA had higher pain scores during motion (3.4 in PCEA patients vs. 1.9 in CEA patients) [21]. A possible explanation is the speed of background infusion in PCEA, 3 mL/h, and the time it takes for a bolus to work; if the pain at rest is acceptable, it requires some planning by the patient to hit the bolus button long before moving in bed, especially during sleep. CEA patients had a rate varying between 5 and 8 mL/h, which probably provided a better baseline of pain control. Nightingale et al. used the Wessex scale (0–3 scale; see the Results section) and found that the pain scores in motion were lower in PCEA (0.81 in PCEA patients vs. 1.23 in CEA patients) [24]. On a 10-point scale, the values would be comparable to 2.6 (PCEA) vs. 4.1 (CEA).

An important finding is the significant reduction in epidural medication among PCEA patients demonstrated in four studies. The reduction in the need for systemic opioid medication can be an important factor in enhancing safety. The guidelines and protocols by the Enhanced Recovery After Surgery group (ERAS), and the workgroup of Procedure-Specific Recommendations (PROSPECT) stress the importance of opioid-sparing anesthesia in surgery [27,28]. In laparoscopic and robotic-assisted surgery, however, epidural analgesia does not play the same role as in open surgery. The recommended combination with epidural analgesia is multimodal analgesia, with a synergy of additional effects of non-steroidal anti-inflammatory drugs and paracetamol. Dexamethasone can have analgesic effects as well as anti-emetic effects and form

Another current recommendation suggests the use of epidural analgesia in open surgeries involving gynecologic oncology, gastrectomy, radical cystectomy, pancreaticoduodenectomy, colonic surgery, rectal surgery, and esophagectomy [4,29,30,31,32,33,34]. 

Reduction in additional, i.e., manual top-ups, may lead to cost savings depending on hospital logistics around epidural top-ups [19]. This may be offset by the additional costs of PCEA.

A previous study revealed that PCEA procedures require more expensive devices than CEA procedures using standard pumps [35]. An analysis showed that, in terms of top-ups, PCEA patients had a time investment of 16 min per patient versus 56 min per patient for CEA patients [19].

Analysis of the retrieved studies showed that PCEA can increase patient satisfaction. However, a difference could only be shown if the control (CEA) group did not already have a high level of patient satisfaction (>90%). Patients’ feeling of being able to self-administer the pain medication could have contributed to these findings. Our analysis results of safety profiles and side effects, including respiratory depression, hypotension, nausea and vomiting, itching, systemic opioid medication, urine retention, and motor block, are shown in forest plots in the Appendix A, Appendix B, Appendix C, Appendix D, Appendix E, Appendix F, Appendix G. For the comparison of the studies, we did not include follow-up after 24 h. It is possible that the cumulative dosing of opioids may have led to other results in analysis.

A recent Cochrane review demonstrated the benefits of PCEA in obstetrical patients include a reduced amount of epidural medication, improved satisfaction, and a reduction in breakthrough pain [6]. Since only obstetric patients and delivery were investigated, the studies were much more homogenous than the surgical patients we included in our analysis. The physiological mechanism of delivery and labor is characterized by increasing pain with periodic breakthrough episodes, whereas the average postsurgical patient has gradually decreasing pain levels and does not experience periodical breakthrough pain due to contractions. However, PCEA in surgical patients also decreased the requirement for additional top-ups, analogous to the results of employing PCEA in obstetric patients.

Limitations: The overall quality of the studies included was moderate. More importantly, there was heterogeneity in the primary outcome parameter (pain scores, I^2^ 35%). The number of studies and the number of included patients was limited, especially regarding patient satisfaction and top-ups. There was also heterogeneity in patient populations, type of surgery, epidural site, and medication used in the protocol. The specific PCEA regimen employed was heterogeneous, with the common factor being “local anesthetic in the epidural medication”.

There are 26 years between the first and the most recent study, adding to the heterogeneity of the study periods. This heterogeneity cannot properly be assessed using I^2^ and Tau^2^ and warrants caution in the interpretation of the results. A proper meta-analysis is therefore not appropriate, as there are an insufficient number of studies concerning the same type of surgery to allow a good comparison. Our findings are presented in forest plots as Appendix A, Appendix B, Appendix C, Appendix D, Appendix E, Appendix F, Appendix G. A funnel plot is provided in the Appendix A, Appendix B, Appendix C, Appendix D, Appendix E, Appendix F, Appendix G to illustrate the extent of publication bias. The limited number of studies makes it difficult to distinguish chance from real asymmetry. An extra confounding factor involves the administration of co-analgesics (paracetamol and metamizole), recorded in two studies (Silvasti and Maca). Maca excluded patients who received systemic medication, whereas Silvasti did not exclude these patients. The other studies neither mentioned nor denied the administration of paracetamol or NSAIDs. We acknowledge this as an unknown (confounding) factor, as it may have influenced the pain scores if one group had received more analgesics than the other group.

## 5. Conclusions

The differences in pain scores between PCEA and PCA were not clinically meaningful. PCEA in surgical patients had advantages over CEA in regard to the amount of epidural drug use, the amount of required rescue systemic analgesics, patient satisfaction, and the number of top-ups required. Further research should evaluate PCEA using more composite postoperative comfort scores instead of primarily focusing on pain scores.

## Figures and Tables

**Table 1:** Basic characteristics of included studies.

Author Year	Study Type	N: PCEA/CEA	ASA	Age: PCEA /CEA	Surgery	Epidural Insertion	PCEA Medication Speed Bolus Lockout	CEA Medication, Speed	Rescue Opiates	Co Analgesics	Pain Service
Nolan 1992 [16]	RCT, PB	11/12	1–2	33/35	Pelvic	L2-4	Bup 0.125% + Fen 1 µg/mL; 4 mL/h; BOL 3 mL 15 min	Bup 0.125% + Fen 1 µg/mL 10 mL/h	NR	NR	APS
Lubenow 1994 [17]	RCT	31/31	1–3	57/60	Thoracic/General/Urologic/Orthopedic/Gynecological	NR	Bup 0.1% + Fen 10 µg/mL; 5 mL/h BOL 1 mL; 10 min	Bup 0.1% + Fen 10 µg/mL 5 mL/h; increase by nurse.	Morphine 1–2 mg/2 h	NR	APS
Hering 1997 [21]	RCT	15/15	1–3	62/63	Major abdominal	T10-L4	Bup 0.125% + Suf 1 µg/mL +clonidine 3 µg/mL 3 mL/h BOL 5 mL; 20 min	Bup 0.125% + Suf 1 µg/mL +clonidine 3 µg/mL; 5–8 mL/h	No	NR	NR
Silvasti 2001 [22]	RCT, DB	26/23	1–3	71/74	Total knee arthroplasty	L2-L3	Bup 0.11% + Fen 5 µg/mL; 0.1 mL/h; BOL 0.05 mL/kg; 3×/h Lockout 10 min	Bup 0.11% +Fen 5 µg/mL; 0.1 mL/kg/h BOL 0.2 mL, Lockout 10 min	Oxy 0.15 mg/kg im.	Paracetamol 3 × 1 g/d.	APS
Nightingale 2007 [24]	RCT	104/101	NR	68/69	Colonic resection	TH	Bup 0.125% + Fen 4 µg/mL; 8 mL/h; BOL 3 mL; 20 min	Bup 0.125% + Fen 4 µg/mL; 15 mL/h	NR	NR	APS
Maca 2018 [20]	RCT	55/56	1–3	66/70	Total hip replacement	L2-L3	Lbup 0.1% + Suf 1 µg/mL; 3 mL/h; BOL 4 mL; 20 min	Lbup 0.1% + Suf 1 µg/mL, 5 mL/h Bolus: 8 mL by physician	Tramadol	Paracetamol Metamizole	ICU

Abbreviations: RCT = randomized controlled trial; PB = patient-blinded; DB = double-blinded; PCEA = patient-controlled epidural analgesia; CEA = continuous epidural analgesia; NR = not reported; TH = thoracic; ASA = American Society of Anesthesiologists; Bup = bupivacaine Lbup = levobupivacaine; Ropi = ropivacaine; Fen = fentanyl; Suf = sufentanil; Oxy = oxycodone; Lockout = lockout period; BOL = bolus dose; ICU = hourly evaluation at intensive care unit.

**Table 2:** Outcome parameters of included studies.

Author	Primary	24 h Pain Score	24 h Pain Score	Mean (sd) PCEA/CEA	*p* Value	Side	Patient	Other	
Year	endpoint	resting mean	motion	epid.drug amount	epid drug	effects	satisfaction		PCEA/CEA
		(sd/Range)	P	[time]					
		P							
Nolan	Pain score	2.6 (0.4–7.2)/1.4 (0–7.8) Range	*	316 (34)/341 (41)	NS	NS	NS		
1992 [16]	VAS (0–10)			Bupivacaine mL					
		NS		[24 h]. SEM					
Lubenow	Pain score	2.7(2.9)/4.2(2.3)	*	219 (140)/307 (80)	<0.01	NS	NR	Mean mg of iv	
1994 [17]	VAS (0–10)	sd		Bupivacaine mL				morphine/patient	
		*p* < 0.05		[48 h]					
								0.16 (0.9)/3.45 (7.7)	
								*p* < 0.05	
Hering	Pain score	0.4 (0.4)/0.4 (0.5)	3.4 (1.1)/1.9 (1.1)	112 (33)/135 (20)	<0.01	NS	93%/100%		
1997 [21]	VAS (0–10)	sd	*p* < 0.01	Bupivacaine mL			NS		
		NS		[24 h]					
Silvasti	Pain score	2.8 (0–6.8)/2.4 (0–6.0) Range	3 (0–10)/4.6 (0–8)	74 (24)/124 (20)	<0.001	NS	NS	VAS (0–50)	
2001 [22]	VAS (0–10)		Range	Bupivacaine mL				recalculated to 1–	
		NS	NS	[20 h]				10 score	
Nightingale	Pain score	0.3 (0.8)/0.54 (0.8) ^	0.81 (0.6)/1.23 (0.7) ^	NR	-	NR	76/43%	Top-ups:	
2007 [24]	*VRS (0–3)*	sd	*p* < 0.001				*p* < 0.0001	13%/36%	
		*p* < 0.05							
								*p* = 0.0002	
Maca	Pain score	1.1 (0.6)/1.2 (0.4)	1.1 (0.6)/1.2 (0.4)	0.9 (0.3)/1.3 (0.4)	<0.001	NR	4.3 (1)/2.8 (0.7)	Satisfaction in 0–5	
2018 [20]	VAS (0–10)	sd	sd	ml/kg/d			*p* < 0.001	Likert scale, higher	
		NS	NS					is better.	

PCEA = patient-controlled epidural analgesia; CEA = continuous epidural analgesia; NS = no significant difference; VAS = visual analog scale; VRS = verbal rating scale; NR = not reported; SEM = standard error of mean is reported instead of sd. * Not specified if pain scores were measured in rest or movement; ^ = mean Wessex pain score (0–3 scale).

**Figure A4:**
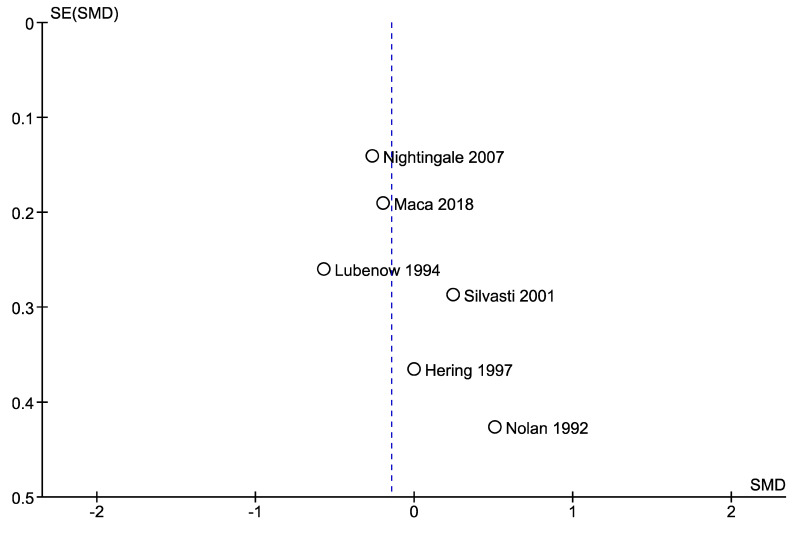
Funnel plot of studies: pain scores in PCEA and CEA. Hering [21], Lubenow [17], Maca [20], Nightingale [24], Nolan [16], Silvasti [22].

## Appendix A. Search Strategy

PubMed

(“Analgesia, Epidural”[Mesh]. OR “Anesthesia, Epidural”[Mesh]. OR epidural an*[tiab]) AND (“Analgesia, Patient-Controlled”[Mesh]. OR patient controlled[tiab]. OR PCEA[tiab]. OR intermittent bolus[tiab]. OR intermittent epidural bolus[tiab]. OR alternative analgesic technique*[tiab]. OR other analgesic technique*[tiab]) AND (continuous epidural*[tiab]. OR continuous infusion*[tiab]. OR epidural analgesia[ti])

EMBASE (Ovid)

**#**	**Searches**
1	epidural analgesia/ or epidural anesthesia/or epidural an*.ti,ab,kw.
2	patient controlled analgesia/ or (patient controlled or PCEA or intermittent bolus or intermittent epidural bolus).ti,ab,kw. or ((alternativ* or other) adj analgesic technique*).ti,ab,kw.
3	continuous epidural anesthesia/ or (continuous epidural* or continuous infusion*).ti,ab,kw. or epidural analgesia.ti.
4	1 and 2 and 3
5	limit 4 to conference abstract status
6	4 not 5

Cochrane Library

ID Search Hits

#1 MeSH descriptor: [Analgesia, Epidural]. explode all trees

#2 MeSH descriptor: [Anesthesia, Epidural]. explode all trees

#3 (epidural an*):ti,ab,kw

#4 #1 or #2 or #3

#5 MeSH descriptor: [Analgesia, Patient-Controlled]. explode all trees

#6 (patient controlled or PCEA or intermittent bolus or intermittent epidural bolus or alternative analgesic technique* OR other analgesic technique*):ti,ab,kw

#7 #5 or #6

#8 (continuous epidural* or continuous infusion*):ti,ab,kw #9 #3 and #7 and #8

## Appendix B. Data Extraction Form

Author	
Year of publication	
study type and blinding	RCT, Patient blinded, Double blinded, Cohort
Number of participants: PCEA/CEA	
ASA categories	1, 2, 3, 4
Age distribution: PCEA/CEA	
Surgery type	Orthopedic, Thoracic, upper abdominal, lower abdominal, pelvic, general, urologic, major abdominal, gynecological, total knee arthroplasty, colonic resection, breast, vascular, total hip replacement
Epidural level	level of epidural catheter insertion
Patient controlled epidural analgesia	
medication, speed,	
Bolus (mg/mL)	
Lock out period	
Continuous Epidural Analgesia	
medication	
speed	
Rescue opiates	
Co analgesics	
Pain Service	type of pain service
Primary endpoint	Pain score (types: visual analog scale, Numeric Rating scale
24 h Pain score resting, mean (sd), SEM, IQR.	
*p* Value	
24 h Pain score in motion	
*p* Value	
Mean(sd)pcea/cea epid.drug [time]	
*p* Value	
Side effects (nausea, pruritus, hypotension, motor weakness)	
Patient satisfaction	
Other	

## Appendix F. Funnel Plot of Studies: Pain Scores in PCEA and CEA Patients

X-axis: left of 0, favoring PCEA; on the right, favoring CEA. The dotted line shows the estimated effect, not in favor of PCEA or CEA. Y-axis: standard error of effect estimate, higher weighted studies at the top. Plot asymmetry is indicative of heterogeneity (study sizes and intervention effects).

## Appendix G. A Summary of Findings (GRADE Table)

**Summary of findings:**						
**PCEA compared to CEA for adult surgical patients**						
**Patient or population**: adult surgical patients **Setting**: **Intervention**: PCEA **Comparison**: CEA						
**Outcomes**	**Anticipated Absolute Effects *** **(95% CI)**		**Relative** **Effect** **(95% CI)**	**№ of Participants (Studies)**	**Certainty of the Evidence (GRADE)**	**Comments**
	**Risk with CEA**	**Risk with PCEA**				
Pain scores assessed with: VAS scale Scale from: 0 to 10 follow-up: mean 24 h	SMD 0.15 lower (0.39 lower to 0.1 higher)		-	480 (6 RCTs)	⨁⨁⨁◯ MODERATE a,b	PCEA reduces pain scores
Top-ups	356 per 1000	134 per 1000 (72 to 237)	OR 0.28 (0.14 to 0.56)	205 (1 RCT)	⨁⨁⨁⨁ HIGH	PCEA results in a large reduction in top-ups.
Not satisfied	468 per 1000	0 per 1000 (0 to 0)	not estimable	284 (3 RCTs)	⨁⨁⨁⨁ HIGH c	PCEA results in a large increase/reduction in satisfaction.
Likert scale outcome Scale from 0 to 5	SMD 1.73 higher (1.29 higher to 2.17 higher)		-	111 (1 RCT)	⨁⨁⨁⨁ HIGH	PCEA results in a large increase in Likert scale satisfaction.
Hypotensive episodes	380 per 1000	415 per 1000 (201 to 668)	OR 1.16 (0.41 to 3.28)	160 (2 RCTs)	⨁⨁⨁◯ MODERATE c,d,e	PCEA increases hypotensive episodes slightly.
Respiratory depression	172 per 1000	89 per 1000 (36 to 201)	OR 0.47 (0.18 to 1.21)	173 (2 RCTs)	⨁⨁⨁◯ MODERATE c,d,f	PCEA results in a reduction in respiratory depression.
Systemic opioids	194 per 1000	111 per 1000 (54 to 218)	OR 0.52 (0.24 to 1.16)	316 (3 RCTs)	⨁⨁⨁⨁ HIGH	PCEA results in a reduction in systemic opioids.
Itching	53 per 1000	70 per 1000 (15 to 264)	OR 1.35 (0.28 to 6.45)	113 (3 RCTs)	⨁⨁◯◯ LOW d	The evidence suggests PCEA increases itching slightly.
Nausea, vomiting	169 per 1000	59 per 1000 (24 to 134)	OR 0.31 (0.12 to 0.76)	273 (5 RCTs)	⨁⨁⨁◯ MODERATE d,g	PCEA likely results in a large reduction in nausea and vomiting.
Urine retention	174 per 1000	17 per 1000 (0 to 253)	OR 0.08 (0.00 to 1.61)	49 (1 RCT)	⨁⨁◯◯ LOW c,h,i	PCEA reduces urine retention slightly.
Motor block	211 per 1000	188 per 1000 (28 to 659)	OR 0.87 (0.11 to 7.24)	79 (2 RCTs)	⨁⨁⨁◯ MODERATE d	PCEA likely results in a reduction in motor block.
Epidural medication	SMD 1.07 lower (1.56 lower to 0.58 lower)		-	273 (5 RCTs)	⨁⨁⨁⨁ HIGH	PCEA results in a large reduction in epidural medication.
* The risk in the intervention group (and its 95% confidence interval) is based on the assumed risk in the comparison group and the relative effect of the intervention (and its 95% CI).						

CI: confidence interval; SMD: standardized mean difference; OR: odds ratio
GRADE Working Group grades of evidence High certainty: We are very confident that the true effect lies close to that of the estimate of the effect. Moderate certainty: We are moderately confident in the effect estimate: The true effect is likely to be close to the estimate of the effect, but there is a possibility that it is substantially different. Low certainty: Our confidence in the effect estimate is limited: The true effect may be substantially different from the estimate of the effect. Very low certainty: We have very little confidence in the effect estimate: The true effect is likely to be substantially different from the estimate of effect.

Explanations

- Unclear risk of random sequence generation (five studies), unclear allocation concealment (three studies), unclear risk of outcome data (four studies);
- Epidural insertion varies from T10 to L4, heterogenous epidural local anesthetics, heterogenous surgery types;
- Small sample size due to a limited number of studies;
- Not always properly described;
- Duration of hypotension not specified;
- Duration not described;
- Nausea and vomiting were treated as a single outcome;
- One study had standard catheterization due to the nature of surgery;
- Catheterization was not always described in all studies.
